# Pioneers of chromosome elimination

**DOI:** 10.3389/freae.2025.1648270

**Published:** 2025-07-29

**Authors:** Luca Comai, Mohan Prem Anand Marimuthu

**Affiliations:** Department of Plant Biology and Genome Center, University of California Davis, Davis, CA, United States

**Keywords:** centromere, CENP-A, plant breeding, evolution, chromosome elimination, missegregation

## Abstract

Epigenetic traits are persistent cellular and organismal properties that do not result from changes in DNA sequence. One such property involves transmission of chromosomes, which entails the formation of highly specialized chromatin structures, the kinetochores, on selected chromosomal regions, called centromeres. Centromere function is essential and centromeres are determined epigenetically by the deposition of a variant histone H3 CENP-A (CENH3 in plants). Either reduced or ectopic function alone leads to genome instability, decreased fitness, aneuploid syndromes, and cancer. At times, however, centromeres malfunction in an apparently programmed mode. This is exemplified by a peculiar centromeric syndrome involving selective elimination of a chromosome set, which can affect a wide range of organisms, including plants. Over half a century ago, plant geneticists described this syndrome in interspecific crosses of barley. Building on their work, we examine the growing understanding of how CENH3 function can be modified to affect epigenetic regulation of centromeres.

## Introduction

During the sexual reproduction of plants and metazoans, the fusion of haploid gametes restores diploidy, thereby maintaining genetic stability across generations. The gametic-zygotic cycle underpins Mendelian inheritance and depends on precise chromosome segregation. Exceptionally, violations of this process occur in developmental and reproductive contexts, where a subset of chromosomes, or even all chromosomes from one of the parents are lost, generating uniparental haploid progeny. Here, we discuss the historical context and advances in plant chromosomal elimination, focusing on barley and arabidopsis.

The discovery of chromosomal elimination did not involve a plant, but the fungus gnat Sciara, in which biased inheritance is driven by parent-specific imprinting of chromosomes (Metz, 1926; Gerbi, 2022). Zygotes with balanced parental chromosomal complements undergo two rounds of chromosome elimination: one in somatic tissues and another in the germline.

In a related phenomenon, crosses between different plant species often yielded uniparental haploids—progeny with the gametic chromosome number and the phenotype of a single parent (Clausen and Mann, 1924; Davies, 1958; Hougas et al., 1958; Kimber and Riley, 1963; Symko, 1969; Kasha and Kao, 1970; Lange, 1971; Ishii et al., 2016). At first, this was explained as “male parthenogenesis”, the development of progeny with a nuclear genome from the male gamete harboured in female cytoplasm. The possibility that, after a normal fertilization, the chromosomes of one parent could be selectively eliminated in the embryo was first discussed in a refereed paper by Symko (1969), who, by rescuing immature embryos, showed high frequency haploid induction in the cross between cultivated barley (*Hordeum vulgare*) and *Hordeum bulbosum.* Interestingly, elimination involved missegregation during embryonic mitosis and depended on parental genome dosage (Kasha and Kao, 1970; Lange, 1971). By the early 1980s, it was clear from cytological investigations (Figure 1), that the uniparental elimination occurred through selective inactivation of the centromeres from *H. bulbosum* (Bennett et al., 1976; Finch, 1983). Finch, specifically, speculated that selective loss of centromeric constriction resulted from epigenetic changes affecting the centromeres of one parent.

**FIGURE 1:**
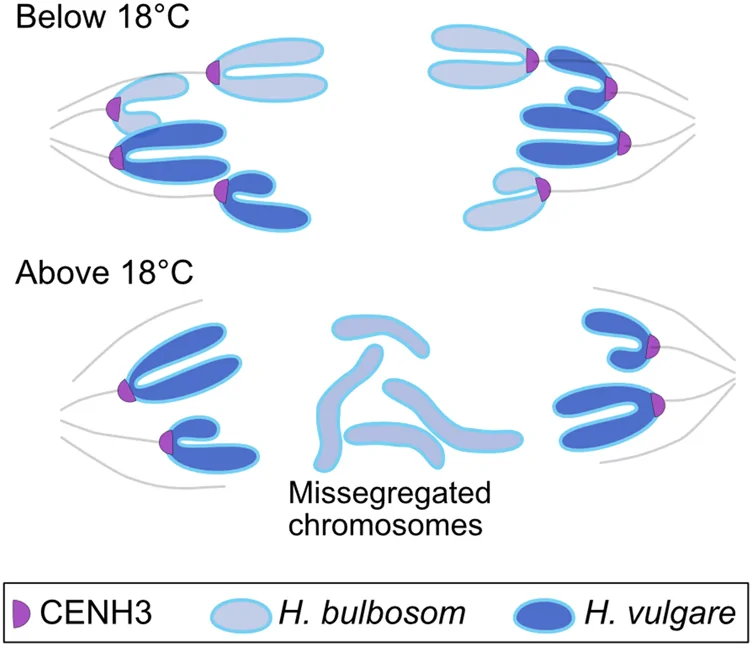
Missegregation of chromosomes depleted of CENH3 chromatin. In the *Hordeum vulgare* x *H. bulbosum* cross, *bulbosum* chromosomes elimination in the embryo is temperature dependent. CENH3 depletion in the missegregating chromosomes (Sanei et al., 2011) demonstrates altered epigenetic regulation, indicating that malfunction is the result of both the hybrid cell environment and temperature interacting with diverged epigenetic states of the centromeres.

These findings had two major implications. First, large numbers of haploids could be generated from interspecific F_1_ hybrids. Upon spontaneous or chemically induced chromosome doubling, these haploids became homozygous recombinant lines—greatly accelerating plant breeding. Second, the system suggested that parental chromosome imprinting could cause postzygotic conflict and selective missegregation.

The molecular basis of this phenomenon remained elusive until two seminal papers highlighted the role of CENH3 in segregation disruption. CENH3 is a variant of histone H3 whose deposition on DNA is both necessary and sufficient to define centromere identity and is essential for chromosome segregation (McKinley and Cheeseman, 2016). Sanei et al. (2011) found that the missegregating *Hordeum bulbosum* chromosomes lost centromeric CENH3, a key discovery linking CENH3 loss to centromeric inactivity (Figure 1). The authors proposed that asynchrony in cell cycle phasing between the two species might prevent CENH3 loading. However, the challenging interspecific nature of the barley system hindered elucidation of the CENH3 behavior. Revealingly, Ravi and Chan (2010) induced uniparental genome elimination through expression of a GFP-tagged CENH3 variant. Using isogenic arabidopsis strains, they demonstrated the unambiguous potential of CENH3 in inducing epigenetic mismatch that causes selective loss of one parental genome in the progeny.

Subsequent research demonstrated that diverse modifications—single and multiple amino acid substitutions, small deletions (Karimi-Ashtiyani et al., 2015; Kuppu et al., 2015; 2020), and even natural variation (Maheshwari et al., 2015)—could all trigger haploid induction in Arabidopsis. Recapitulating interspecific crosses, outcrossing CENH3-modified plants to the wild-type triggers epigenetic incompatibility. Presumably, centromeres with weak CENH3 were selectively eliminated in a postzygotic competition.

How exactly this competition unfolded was unclear, particularly because CENH3 was thought to be stripped from chromatin in the zygote, as shown with a non-functional CENH3 variant (Ingouff et al., 2010). If CENH3 is removed, what preserves centromere identity across generations? The answer emerged with the discovery that only the altered version, but not the wild-type CENH3, is removed from centromeres in the mature egg cell (Marimuthu et al., 2021). Removal may depend on an active cellular surveillance mechanism (Mérai et al., 2014) and is likely sensitive to temperature and methylation changes (Marimuthu et al., 2021; Wang et al., 2023). In the hybrid zygote, as a consequence of this differential stability, CENH3 and kinetochore proteins are preferentially loaded onto wild-type centromeres before the first mitosis. This repeats in the following mitoses. A model of cooperative binding explains this phenomenon (Figure 2): in the limited window of the G2 phase, when CENH3 loads, centromeres enriched in CENH3 attract more of it, while depleted ones are left bereft—“the rich get richer.” As the embryo grows by cell division, chromosomes with weaker centromeres are either lost forming haploids or gradually regain a normal CENH3 load forming aneuploids or maintaining diploidy (Marimuthu et al., 2021). Whether and how this applies to barley’s haploid induction remains to be elucidated.

**FIGURE 2:**
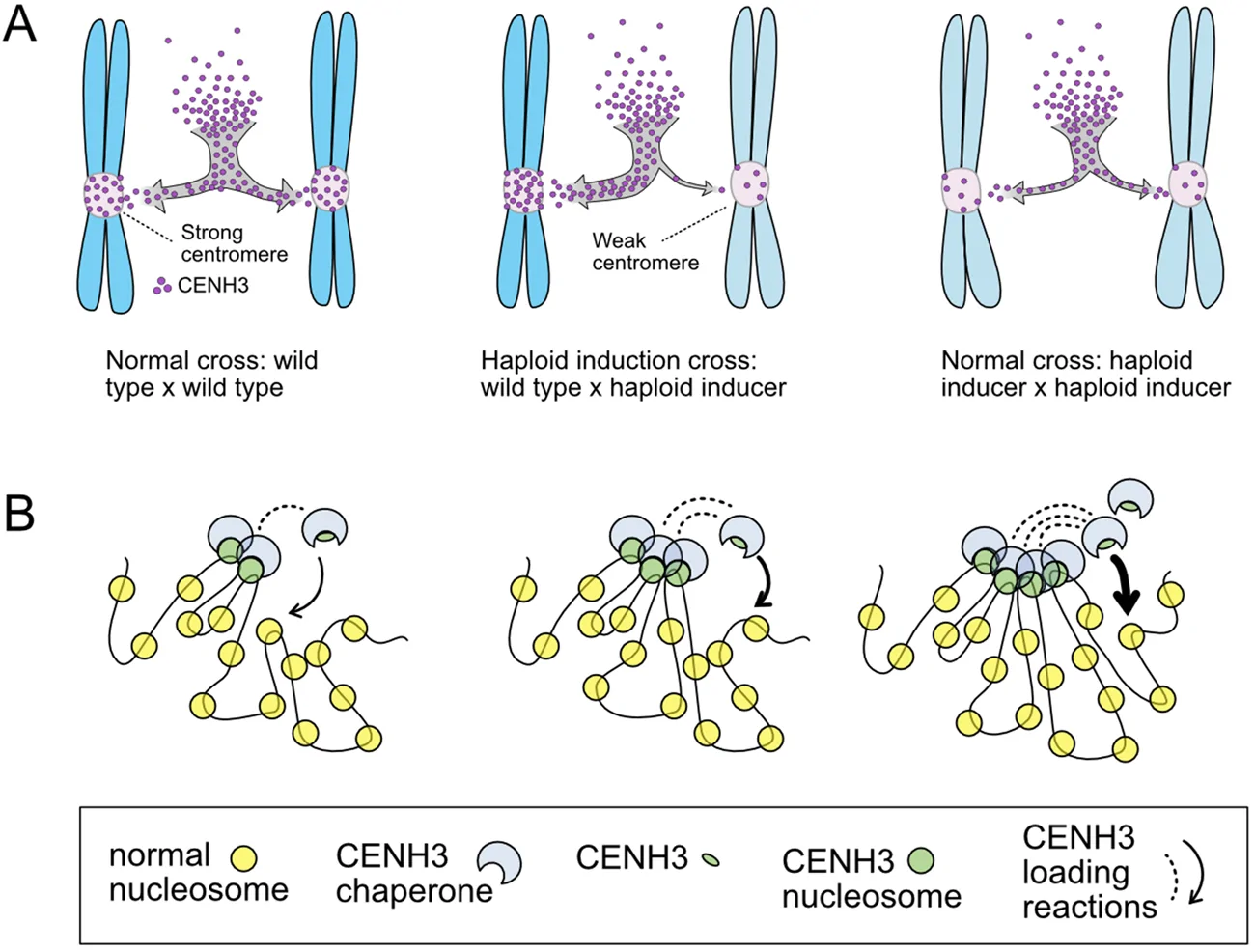
Differential loading of CENH3 and kinetochore components on centromeres with different CENH3 content. The gametes of arabidopsis CENH3-based haploid inducers contribute chromosomes with CENH3-depleted centromeres (Marimuthu et al., 2021) **(A)**. In the zygote, these epigenetically weak centromeres compete poorly with wild-type ones during loading of CENH3 and kinetochore components (not shown) **(B)**. This property can be explained by cooperative binding of new CENH3 to CENH3-chromatin, similar to the model for λ CI repressor function (Johnson et al., 1979). The figure illustrates how centromeric chromatin can organize dispersed CENH3 nucleosomes in interacting superstructures (Fukagawa and Earnshaw, 2014) that could facilitate cooperative behavior potentially through loading cofactors such as KNL2 (Sandmann et al., 2017). The mechanism illustrated could potentially help explain the assembly of homogenous CENH3 domains in plants with two CENH3 types (Ishii et al., 2015; Maheshwari et al., 2017; Karimi-Ashtiyani et al., 2024).

This cooperative binding mechanism also explains a baffling feature of CENH3-based haploid inducers: mutual suppression. Self or cross pollination among various haploid inducers results in normal diploids because two equally depleted centromere sets do not compete and load balanced CENH3 (Figure 2). Indeed, most haploid inducers appear phenotypically normal suggesting that an altered CENH3 allele could be fixed in a population where it would act as a barrier to outcrossing. Even if it appears viable, a weaker CENH3 could have at least two deleterious consequences: i) increased missegregation due to decreased probability of spindle capture; ii) decreased centromeric stability because of decreased dominance over emerging neocentromeres, which start as sites with lower CENH3 density. Therefore, selection against CENH3 with suboptimal loading efficiency is likely. Direct experimental evidence for these predictions, however, is needed.

The demonstration of efficient CENH3-based haploid production in arabidopsis spurred efforts to transfer this technology to crops. The method entails complementation of a null *CENH3* allele with a gene expressing a fusion protein or a mutant protein. The rules for complementation and haploid induction, however, have turned out to be variable and even capricious. The CENH3 GFP-tailswap fusion, the best haploid inducer modification in arabidopsis (Ravi and Chan, 2010; Kuppu et al., 2020), failed to complement the CENH3 knockout in tomato and maize (Kuppu et al., 2020; Wang et al., 2021). Other CENH3 fusions were competent at complementation, but failed to induce haploids (Meyer et al., 2023). Success was achieved through related approaches (Lv et al., 2020; Wang et al., 2021; Manape et al., 2024). Engineered depletion of CENH3 in the egg cell via proteasome targeting may simplify translation of the technology (Somasundaram et al., 2025). Notably, heterozygosity alone can result in haploid induction: a −/+ genotype resulted in 5% haploid induction rate in maize (Wang et al., 2021), but only 0.5% in arabidopsis (Marimuthu et al., 2021). Heterozygosity for a null allele (−) works because mitotic divisions in the (−) gametophyte result in progressive CENH3 depletion from the centromere. If the (−) egg fuses with a (+) sperm, the depleted, epigenetically weak centromeres are outcompeted by the wild-type ones. The different efficiency of the same approach in different species is probably the result of variation in CENH3 and its protein interactors. Could different centromeric sequences also play a role?

The specificity of centromeric DNA interaction with CENH3 remains mysterious. Following targeted or accidental CENH3 deposition, neocentromeres can nucleate on diverse DNA sequences (Fu et al., 2013; Dawe et al., 2023). CENH3 of one species can function with centromeres of another (Talbert et al., 2002; Comai et al., 2017; Maheshwari et al., 2017; Karimi-Ashtiyani et al., 2024). At the same time, genome-wide homogenization of centromeric repeats suggests optimization (Maheshwari et al., 2017; Altemose et al., 2022; Wlodzimierz et al., 2023) as does the evolution of CENP-B and its target sequence in certain mammals (Gamba and Fachinetti, 2020). It remains to be elucidated, however, whether the interaction between CENH3 and centromeric repeats drives their accelerated evolution (Henikoff et al., 2001). In support of this hypothesis, stronger centromeres result in meiotic drive (Chmátal et al., 2014; Akera et al., 2019). Notably, CENH3-occupied regions were directly associated with the *de novo* integration of certain transposons, demonstrating a specific link between CENH3 chromatin and centromere evolution (Tsukahara et al., 2025). CENH3 mutations could suppress meiotic drive caused by preferential binding of CENH3 to certain repeats (Henikoff et al., 2001; Finseth et al., 2021; Finseth, 2023), but direct binding evidence is missing.

In conclusion, localization of CENH3/CENP-A on centromeric DNA is self-determining, likely constituting a positive forward loop similar to CI of λ phage (Johnson et al., 1979). This epigenetic interaction is subject to several constraints: suppression of competitive sites; effective maintenance through rapid and specific loading before mitosis and meiosis; and fruitful interaction with the kinetochore complex. The balancing of these requirements likely results in evolutionary variations that, as observed by our epigenetic trailblazers when genetically diverged organisms are crossed, lead to conflict and whole-genome failure of a centromere set.

## Data Availability

The original contributions presented in the study are included in the article/supplementary material, further inquiries can be directed to the corresponding author.
